# Supplementary material for: Are carbon emissions trading and green financial instruments synergistic? -Comprehensive quantitative research based on content analysis
Source: PLoS One. 2024 Mar 7;19(3):e0298601. doi: 10.1371/journal.pone.0298601 (PMC10919601; doi:10.1371/journal.pone.0298601)
Supplement: S1 Data — (ZIP) [file pone.0298601.s001.zip › Data/地级市Z_F工作报告.zip!/╜¡╦╒/╦╒╓▌╩╨/└┤╫╘╣1⁄2╓┌║┼í╛┬φ┐╦╩2╛▌═°í┐_2015.docx]

各位代表：

　　现在，我代表苏州市人民政府，向大会报告工作，请予审议，并请市政协各位委员和其他列席人员提出意见。

一、2014年工作回顾

　　过去的一年，在中共苏州市委的正确领导下，我们认真学习贯彻中共十八大、十八届三中四中全会和习近平总书记系列重要讲话精神，团结依靠全市人民，牢牢把握稳中求进工作总基调，主动适应经济发展新常态，全面深化重要领域改革，统筹做好产业升级、创新驱动、扩大开放、城乡一体、环境优化、文化繁荣、法治建设、社会治理、民生改善等各项工作，推动了经济社会持续健康发展。

　　1.经济运行稳中提质。全市预计完成地区生产总值1.35万亿元，增长8%；地方公共财政预算收入1443.8亿元，增长8.5%；全社会固定资产投资6230.7亿元，增长3.8%。工业经济平稳运行，实现规模以上工业总产值3.06万亿元，新兴产业产值、高新技术产业产值所占比重分别提高到47.5%和44.8%。推动信息化和工业化“两化”融合，大力发展智能制造，支柱产业提档升级步伐加快。服务业量质齐升，实现增加值6305亿元，增长9%，占地区生产总值的比重达到46.7%。国家现代服务业综合试点工作积极推进，总部经济和新兴业态快速发展。新增各类金融机构111家，金融总资产达到3.4万亿元，增长13.3%。完成社会消费品零售总额4062亿元，增长12%；电子商务交易额超过5000亿元，增长40%。实现旅游总收入1675亿元，增长10%；姑苏区被命名为首个国家古城旅游示范区，苏州高新区镇湖国家级生态旅游示范区创建工作通过验收。文化产业主营业务收入达到3565亿元，增长15%；中国工艺文化城入选国家特色文化产业重点项目。农业现代化水平保持全省领先，“四个百万亩”布局得到优化，新增现代农业园区面积9千公顷、高标准农田面积3.2千公顷，农业机械化水平达到87.5%。农产品质量安全稳定可控，又添绿色、无公害、有机农产品126个。粮食生产喜获丰收，地方储备粮三年扩储任务顺利完成，我市成为国家粮安工程建设试点示范市。

　　深入实施创新驱动战略，科技综合实力不断增强。持续增加研发投入，全社会研究与试验发展经费支出占地区生产总值的比重达到2.7%。新认定国家高新技术企业669家，新增省级以上工程技术研究中心101家、工程中心（实验室）10家、企业技术中心65家，新加坡-中国（苏州）创新中心落户苏州工业园区，中国POWER技术产业生态联盟在苏州高新区成立。苏州自主创新广场获批国家技术转移苏南中心，苏州高新区成为全国科技创新服务体系建设试点单位。积极拓展产学研合作，我市与中国科学院签署新一轮深化院市合作备忘录。促进科技与金融深度融合，扩大科技信贷风险补偿资金扶持范围，全年帮助中小科技企业获得信贷资金31亿元。专利申请量、授权量分别达到10.5万件和5.3万件，万人有效发明专利拥有量达到18.2件。我市国家城市综合服务标准化试点通过评估，张家港市成为国家知识产权示范城市。国际精英创业周、“赢在苏州”海外系列创业大赛成效明显，中国苏州人力资源服务产业园建设取得新成果。新增国家“千人计划”人才32人，累计达到157人，其中创业类人才95人，继续位居全国城市首位；新增省“双创计划”人才98人，累计达到501人，连续八年名列全省第一。全市人才总量达到210万人，其中高层次人才15.5万人，高技能人才46.6万人。

　　2.改革开放持续深化。加快行政审批制度改革，削减行政审批事项294项，市级机关不再保留非行政许可审批类别。市级行政审批事项目录清单、政府行政权力清单、行政事业性收费目录清单、政府部门专项资金管理清单和市级政府性基金管理清单向社会公布。启动行政审批服务中介机构改革。简化企业设立审批流程，实行企业设立登记“一口受理、一口办理”。工商注册资本登记制度改革成效显著。制定政府向社会转移职能事项目录，推进政府向社会购买服务。新一轮政府机构改革任务基本完成。经济发达镇行政管理体制改革试点深入开展。国有企业分类监管不断加强，市属国资公司混合所有制改革试点启动实施。民营经济健康发展，私营企业达到29.6万户，增长15.6%；注册资本1.15万亿元，增长18.9%。学习借鉴中国（上海）自由贸易试验区成功经验，31项改革政策在我市复制推广。苏州工业园区获批开展贸易多元化试点和4项跨境人民币创新业务试点。昆山深化两岸产业合作试验区金融创新步伐加快，跨境人民币双向借款突破100亿元。昆山市被国家列为两岸冷链物流产业合作试点城市。苏州金融资产交易中心获批筹建，苏州高新区全国股转系统首家委托服务机构揭牌运营。新增上市企业8家、“新三板”挂牌企业71家。

　　对外贸易保持增长，开放型经济提质增效。完成进出口总额3113.1亿美元，增长0.6%，其中出口1811.8亿美元，增长3.1%。外贸结构持续优化，一般贸易进出口比重达到29.7%，比上年提高2.5个百分点；加工贸易转型升级步伐加快，“苏州造”内外贸一体化网上销售平台建成运营。服务外包接包合同额、离岸执行额分别增长20.4%和18%。“苏满欧”五定班列实现常态化运营，太仓港纳入启运港退税政策试点。实际使用外资81.2亿美元，其中服务业使用外资占到37.6%，新兴产业和高技术项目使用外资占到47%。跨国公司总部外汇资金集中运营管理试点范围进一步扩大，资金池规模达到66亿美元。海关特殊监管区外汇监测平台推广运用。中方境外协议投资额达到17亿美元，增长5%，总量实现全省“十一连冠”。中新联合协调理事会第16次会议在我市成功召开。相城经济开发区升格为国家级经济技术开发区，太仓港综合保税区通过国家验收。南北共建合作进一步深化，苏州宿迁工业园、苏州南通科技产业园、苏州盐城沿海合作开发园区再创新成果。对口支援西藏林周和新疆霍尔果斯、巩留、阿图什，以及对口帮扶贵州铜仁等工作扎实开展。积极参与“一带一路”建设，抓住丝绸之路沿线国家元首访苏机遇，有效促进经贸文化交流。新增国际友城2对，累计缔结48对。对台合作全面深化，交流领域不断拓宽。侨务工作得到加强，新增2个全国社区侨务工作示范单位。

　　3.城乡发展更趋协调。新一轮苏州市城市总体规划修编前期工作积极开展，一批分区规划、专项规划、详细规划和城市设计相继制定。国家历史文化名城保护工作得到加强，东部综合商务城、西部生态科技城、南部太湖新城和北部高铁新城建设呈现新亮点。沪通铁路苏州段如期开工，常嘉高速公路昆山至吴江段、张家港疏港高速公路和312国道苏州西段改扩建等工程抓紧建设。中环快速路及市区内环快速路延伸线局部主线建成通车，新建、改造一批城市道路。轨道交通2号线延伸线、4号线及支线工程加快实施。苏州高新区有轨电车1号线建成通车。内河港口发展三年行动计划顺利实施，港口建设有序推进，苏州港集装箱吞吐量达到445万标箱。苏南运河苏州段四级航道改三级航道和申张线、杨林塘航道整治工程扎实推进。特高压锦苏线引入西南水电328亿千瓦时。建设智慧城市，4G 网络覆盖全市，苏州市、昆山市入选“宽带中国”示范城市，我市成为全国下一代互联网示范城市。数字城市地理空间框架基本建成，地理国情普查全面开展。新增人防工程102万平方米。城郊结合部、城中村、棚户区、老旧小区、背街小巷、城市河道、低洼易淹易涝片区、建设工地、农贸市场综合整治成效明显，内环快速路沿线整治任务如期完成。调整城市管理体制，基层属地管理责任和执法力量得到加强，“数字城管”实现全覆盖。

　　抓住我市被列为国家城乡发展一体化综合改革试点的机遇，大力实施三年行动计划，城乡规划、现代农业、节约集约、强村富民、美丽镇村、工农互惠水平进一步提高。农村集体经济持续壮大，集体资产总量、村均稳定性收入、社区股份合作社分红均增长10%以上。社区股份合作社股权固化改革稳步实施，农村土地承包经营权确权登记颁证试点工作有序开展。家庭经营、集体经营、合作经营、企业经营加快发展，现代农业经营体系逐步建立。推广“农发通”等金融产品，扩大农业担保规模，拓展农业保险领域，农村金融服务能力不断增强。疏浚整治一批农村河道，圩区堤防达标水平得到提升。大力建设美丽镇村，示范镇综合提升工程进展顺利，创建美丽村庄示范村10个、三星级康居乡村101个，农村人居环境持续改善。

　　4.生态建设力度加大。制定生态红线区域保护规划和主体功能区实施意见，划定11大类、103个生态红线区域。推进生态文明建设“十大工程”，抓好年度79个重点项目，完成投资238亿元。严格保护水源水质，集中式饮用水水源地水质达标率保持100%。加强水环境治理，东太湖综合整治一期工程和古城河道、石湖水质提升工程顺利完成，阳澄湖生态优化、七浦塘拓浚整治等工程实现年度目标。完成大气污染防治重点项目254项，淘汰燃煤锅炉438台、老旧机动车7.9万辆，推广纯电动汽车1200辆，PM　年均浓度下降6.3%。防控噪声污染，声环境功能区噪声达标率保持100%。重金属污染治理和土壤修复工作积极开展。优化生活垃圾收运处理，市区新建改建垃圾转运站16座，新增生活垃圾分类试点小区197个。狠抓节能减排，单位地区生产总值能源消耗、主要污染物排放总量削减完成省下达任务。落实“关停不达标企业、淘汰落后产能、改善生态环境”三年专项行动计划，淘汰、关停落后企业1255家。做好国家低碳城市试点工作，开展万家企业节能低碳行动，苏州工业园区成为国家首批低碳试点园区。常熟经济技术开发区获批国家生态工业示范园区，张家港再制造产业示范基地成为国家循环化改造示范试点园区。严格执行基本农田保护制度和耕地占补平衡制度，土地节约集约利用水平进一步提高。上方山石湖生态园、虎丘湿地公园、吴中区临湖园博园、“两河一江”、“两山一镇”等工程扎实推进。市区新增绿地505万平方米，农村新增林地绿地3.6千公顷，全市陆地森林覆盖率达到29.4%。加强湿地保护和管理，吴江同里湿地、昆山天福湿地成功创建国家湿地公园。贯彻《苏州市生态补偿条例》，补偿机制不断完善。

　　5.社会建设成效明显。坚持教育优先发展，推进教育现代化。有序开展招生考试、教育评价制度改革，试点实行校长职级制，创新教师培训模式，促进教师校际交流。新建小学、幼儿园37所，完成校舍安全工程121万平方米，提档升级外来工子弟学校74所。我市被评为国家中小学安全教育实验区。建成苏州高等幼儿师范学校迁建主体工程。成立全省省辖市首家特殊教育指导中心。昆山杜克大学招生开课，世界联合学院分院落户常熟市。成功承办首届世界语言大会。巩固提升国家公共文化服务体系示范区建设成果，文化惠民成效明显。建成中国昆曲剧院，设立全国首个轨道交通图书馆。建设“书香苏州”，在全国首创推出“网上借阅·社区投递”便民服务。繁荣文艺创作，一批优秀文学、艺术作品分获全国“五个一工程”奖、鲁迅文学奖、曹禺剧本奖和中国曲艺牡丹奖。新增专题博物馆、美术馆11座，苏州博物馆吴门画派·文徵明特展获得全国博物馆陈列展览精品奖。大运河苏州段成功列入世界文化遗产，新增国家级历史文化名镇3个、名村3个，虎丘地区综合改造和桃花坞历史文化片区、平江历史街区、石湖渔家村越城遗址、同里历史文化遗产保护整治等工程取得新进展，可园、柴园等古典园林整体修复继续推进。我市成功入选联合国教科文组织“全球创意城市网络-手工艺与民间艺术之都”，国家级非物质文化遗产生产性保护示范基地（苏绣）落户苏州高新区。《苏州市志（1986～2005）》出版发行。加强医疗和公共卫生服务体系建设，基本建成“15分钟健康服务圈”。县级公立医院综合改革继续深化，药品加成全面取消。修订医疗机构设置规划，鼓励社会资本办医。苏州大学附属第一医院主体迁建一期工程如期完成，苏州大学附属儿童医院园区总院基本建成。医疗自助服务进家庭和婴幼儿健康促进工程取得成效。一方是独生子女的夫妇可生育两个孩子的政策顺利实施。市民健身中心体育场、体育馆按期建成。在第18届省运会上，我市代表团获得的金牌总数、奖牌总数均首次位居全省第一。成功举办首届市民健身运动会。民族宗教工作创新开展。妇女儿童发展规划深入实施。加强国防教育和国防后备力量建设，推动军民融合深度发展，认真落实优抚安置政策，创建省双拥模范城实现“满堂红”。

　　坚持把社会主义核心价值观贯穿于各类文明创建活动，社会文明程度进一步提升。哲学社会科学繁荣发展，全民科学素质行动计划深入实施。民主法治创建取得新成效，国家级、省级民主法治示范村（社区）数量保持全省领先。创新开展法治宣传教育，我市被评为全国“六五”普法中期考核先进市。积极构建“半小时法律服务圈”，公共法律服务体系加快建设。法律援助服务标准化试点深入开展。社区矫正规范化建设持续加强。创新社会治理，“政社互动”试点实现全覆盖。培育发展社会组织，我市获评全国社会组织建设创新示范区。推行依法逐级走访和诉访分离，加大矛盾纠纷排查和信访积案化解力度，一批疑难复杂纠纷得到解决。市公共信用信息服务平台一期工程和企业征信服务平台建成运行。推进平安中国示范区建设，加强社会治安综合治理，依法打击违法犯罪活动，圆满完成国家重要活动期间的重大安保维稳任务。昆山中荣公司“8·2”特别重大事故发生后，迅速学习贯彻习近平总书记和李克强总理重要批示精神，严格落实党中央、国务院和省委、省政府要求，全力做好事故救援、伤员救治和后续处置等工作。深刻吸取血的教训，进一步强化安全生产责任，全面开展粉尘爆炸危险企业专项整治和“六打六治”打非治违行动，狠抓重大隐患挂牌整改和安全生产网格化监管，最大程度消除安全隐患和不稳定因素。完善应急救援指挥平台，组建水上搜救应急处置中心和电梯应急救援指挥中心，建成应急物资储备库、救援专家库和专业队伍库，提高应急管理能力。

　　6.人民生活不断改善。认真办好民生实事，持续增加民生投入，城乡公共服务支出占公共财政支出的比重达到76.7%。城镇居民人均可支配收入达到4.66万元，农村居民人均可支配收入达到2.36万元，分别增长8.5%和10%。新增就业17.2万人，开发公益性岗位1.15万个，免费培训城乡劳动者43.6万人，苏州籍高校毕业生就业率达到98.4%，城镇登记失业率控制在2%，“零就业”、“零转移”贫困家庭保持动态清零。实施促进创业带动就业政策，新建创业孵化载体12个，累计达到173个，苏州市大学生公共实训基地获批国家级创业孵化示范基地。城镇职工社会保险覆盖率、城乡居民养老保险和医疗保险覆盖率均保持在99%以上，居民医疗保险财政补助标准由每人每年310元提高到360元。城乡老年居民全部纳入社会化管理服务。最低生活保障标准由每人每月630元提高到700元。低保边缘重病困难对象救助范围进一步拓宽，医疗费用自负部分救助比例由60%提高到70%。“苏银通”业务开通运行，市区养老金实现免费跨行存取。新增养老床位6125张、日间照料中心96个、助餐点163个，市怡养老年公寓建成启用。残疾人社会保障和服务水平进一步提升，发放残疾人各类专项生活救助金1.2亿元。探索建立流动人口积分管理制度，拓展居住证服务功能。开工建设保障性住房46794套，基本建成32773套，公租房和廉租房并轨运行。新增住房公积金缴存职工65.8万人，职工月均缴存额提高7.8%。市区15个老住宅小区、57幢零星居民楼、25条街巷综合整治工程全部完工，12个城中村（无地队）改造完成年度任务；梅巷片区、解放新村片区危旧房改造稳步推进；完成居民家庭天然气置换4万户。国家“公交都市”创建工作全面启动，市区新购新能源和清洁能源公交车600辆，新增出租车500辆。开展食品安全放心行动，强化药品安全综合治理。肉类流通追溯体系覆盖全市，农产品质量安全监管一期平台建成使用，新增放心粮油店（柜）52个。全市新增“菜篮子”保供基地333公顷，市区新建和改造标准化农贸市场10家。加强价格监管，居民消费价格总水平涨幅控制在2.1%。

　　与此同时，我们不断加强政府自身建设，努力为市民提供优质高效服务。紧扣“为民务实清廉”主题，严格按照“照镜子、正衣冠、洗洗澡、治治病”总要求，聚焦反对“四风”，认真开展党的群众路线教育实践活动。学习弘扬焦裕禄精神，自觉践行“三严三实”要求，坚决执行改进作风、密切联系群众的各项规定，狠抓群众提出的意见建议和中央巡视组反馈意见的整改落实，政府机关作风有了明显转变。学习贯彻中共中央关于全面推进依法治国若干重大问题的决定和省委制定的贯彻落实意见，推动依法行政和法治政府建设。市政府提请市人大常委会审议通过地方性法规1件，颁布政府规章3件、规范性文件6件，并对10件政府规章和2件规范性文件进行立法后评估。健全行政执法与刑事司法衔接机制，完善行政执法网上监督平台。深化政务公开，建立重大行政决策网上公开运行系统，市政府预决算、市级部门预决算和市级“三公”经费预算全部公开。全市性政府会议减少16%，市政府文件精简35%，清理评比达标表彰项目114个，市级因公出国（境）、公务用车购置及运行、公务接待经费下降20.1%。严格落实党风廉政建设责任制，推进政府系统惩治和预防腐败体系建设，强化监督执纪问责，严惩腐败行为，反腐倡廉取得新成效。深化财政预算执行审计，强化“三责联审”，加强重点公共工程跟踪审计，提高财政资金使用效率，监督权力运行，服务民生改善。我市审计工作再次被评为全国先进。圆满完成第三次经济普查任务，并获国家先进集体称号。市政府自觉接受市人民代表大会及其常委会的法律监督、工作监督和市政协的民主监督，坚持重大事项向市人大常委会报告、向市政协通报，注重发挥各民主党派、工商联、无党派人士和人民团体的参政议政作用。组织实施人大议案2件，办复人大代表建议236件、政协提案417件。

　　各位代表，在经济下行压力加大、困难挑战增多、安全生产事故突发的情况下，去年取得的成绩来之不易，这是全市人民团结奋斗、攻坚克难、艰辛努力的结果。在此，我代表市政府向全市人民，向人大代表、政协委员，向离退休老同志，向各民主党派、工商联和无党派人士，向各人民团体，向驻苏解放军、武警和消防官兵，向国家和省驻苏单位，向参与、支持、关心苏州现代化建设的海内外朋友，表示衷心的感谢和崇高的敬意！

　　回顾过去的一年，我们也清醒地看到，前进中还存在许多矛盾和问题，政府工作与人民期待相比仍有差距，主要是：经济增长动力不足，下行压力较大，部分企业生产经营困难；产业结构不够合理，自主创新能力不足，资源环境等瓶颈约束加剧；制约经济社会发展的体制机制障碍依然存在，改革创新任务繁重；公共服务资源配置不够均衡，民生改善还有不少问题需要解决；社会治理仍面临不少新情况、新问题；昆山中荣公司发生了特别重大安全生产事故；政府职能还需加快转变，作风建设、廉政建设、法治建设必须进一步加强。我们一定高度重视这些问题，采取更加有力的措施，切实加以解决。

二、2015年主要任务

　　今年是全面深化改革的关键之年，是全面推进依法治国的开局之年，也是“十二五”收官之年。世界经济虽然可能略有回升，但是总体复苏疲弱态势难有明显改观。我国经济发展进入新常态，苏州正处于转型升级的关键期。去年12月，习近平总书记在视察江苏时作了重要讲话，赋予了江苏“两个率先”光荣使命以新的内涵，明确了努力建设经济强、百姓富、环境美、社会文明程度高的新江苏的最新定位，提出了推动经济发展、现代农业建设、文化建设、民生建设和全面从严治党迈上新台阶的重点任务。我们要认真学习、深刻领会、全面贯彻总书记重要讲话精神，以总书记对江苏工作的总要求作为苏州未来发展的总命题和引领各项工作的总纲领，当好“迈上新台阶、建设新江苏”的先行军和排头兵。

　　根据中共苏州市委十一届八次全体（扩大）会议的部署，今年政府工作的总体要求是：全面贯彻中共十八大和十八届三中、四中全会精神，以邓小平理论、“三个代表”重要思想、科学发展观为指导，以习近平总书记系列重要讲话精神为根本遵循，按照总书记对江苏工作的最新要求，积极适应经济发展新常态，坚持稳中求进工作总基调，以提高经济发展质量和效益为中心，更加注重以创新驱动引领转方式调结构，更加注重统筹城乡发展，更加注重民生改善和权益保护，更加注重生态环境保护，更加注重历史文化传承保护，协调推进全面建成小康社会、全面深化改革、全面推进法治苏州建设、全面从严治党，努力实现经济平稳健康发展与社会和谐稳定。

　　全市经济社会发展的主要预期目标是：地区生产总值增长8%左右，一般公共预算收入增长8.5%左右，固定资产投资6000亿元左右，社会消费品零售总额增长12%左右，进出口总额保持稳定，实际使用外资70亿美元左右，居民人均可支配收入增长高于地区生产总值增幅，居民消费价格总水平涨幅控制在3%左右，城镇登记失业率控制在3.5%以内，全社会研究与试验发展经费支出占地区生产总值的比重提高0.1个百分点，单位地区生产总值能耗下降、主要污染物排放总量削减完成省下达任务。

　　在新的一年里，我们将重点做好以下九个方面工作。

　　1.坚持提质增效，促进经济稳增快转。牢牢抓住转方式、调结构这一关键，推动经济在转型升级中行稳致远，努力实现有质量有效益的持续稳定增长。确保经济稳健运行。发挥政府投资的引导作用，落实鼓励社会投资的政策措施，充分激发市场活力，带动民间资本参与重点领域建设。着力抓好210项重大项目，年内完成投资超过1600亿元，支持新增长点项目和企业技术改造。超前谋划和储备全局性、战略性项目，争取更多项目纳入国家规划。创新消费供给，释放消费潜力，不断满足个性化、多样化的消费需求。加快建设现代商贸集聚区，优化发展社区商业、农村商业。推进国家电子商务示范城市建设，完善电子商务示范基地功能，新增一批电子商务示范平台、示范企业。加大古城旅游示范区和旅游度假区建设力度，扩大休闲度假旅游消费，提高旅游产品品质、旅游服务质量、市场监管水平和旅游业的综合效益。培育文化、信息、养老、健康等潜力大前景好的消费热点，促进大众消费。加强出口基地和出口品牌建设，提高一般贸易出口比重。加快加工贸易转型升级，提升服务贸易集聚化、品牌化水平。保持服务外包快速发展势头，力争接包合同额、离岸执行额均增长15%。扩大先进技术设备、关键零部件和能源资源进口。继续办好中国（苏州）电子信息博览会、中国（昆山）品牌产品进口交易会。强化地方政府性债务管理，有效防控金融、房地产市场和产能过剩等风险，加强对困难企业的风险预警和帮扶解困。调整优化产业结构。发展新产业、新技术、新业态、新模式，推动制造业向智能化、高端化、服务化迈进，打造工业经济升级版。培育壮大高端装备制造、新一代信息技术、节能环保、新材料、生物技术和新医药、新能源等新兴产业，加快形成新的支柱产业，新兴产业产值占规模以上工业总产值的比重提高1个百分点左右。鼓励中小微企业走“专精特新”之路，构建产业链长、附加值高、特色鲜明的产业基地和产业集群。争创国家品牌培育示范城市，形成一批国家级质量标杆和品牌培育示范企业。坚决淘汰落后产能，下大力气化解过剩产能，严控高能耗、高污染、资源性项目和低水平重复建设项目。推动现代服务业跨越发展，力争服务业增加值占地区生产总值的比重提高1个百分点左右。积极利用物联网、云计算、大数据，大力发展平台经济。建设服务业集聚区，培育和引进总部企业做大做强现代物流、信息服务、商务服务、创意设计、检验检测认证等生产性服务业，提升发展生活性服务业。建设中新金融创新合作试验区、台资金融机构集聚区和金融服务外包集聚区、科技保险创新试验区三大金融高地，构建区域金融中心。着力培育文化创意产业，使之与其他产业融合发展，催生新技术、创新新工艺、开发新产品，实现文化产业规模、质态和效益的全面提升。办好第四届中国苏州文化创意设计产业交易博览会。大力推进创新驱动。抓住苏南国家自主创新示范区建设的机遇，集聚国际国内优质创新资源，推动区域创新一体化发展。构建“三核、多区、两中心”的空间布局，着力把苏州工业园区、苏州高新区、昆山高新区打造成为创新驱动的核心区，支持吴江汾湖高新区、常熟高新区升格为国家高新区，建设各县级市（区）省级高新区，做实做优国家技术转移苏南中心、区域性科技金融服务中心。强化企业创新主体地位，围绕产业链部署创新链，围绕创新链完善资金链，加大政策扶持力度，引导企业增加创新投入，撬动社会资金助力各类创新。深入实施“雏鹰”、“瞪羚”计划，促进中小科技企业快速成长。推动科教协同创新，深化产学研合作，争取更多创新研究机构、合作研发项目和重大科技成果落户苏州。完善纳米大科学装置、中科院科技服务网络苏州中心等载体平台功能，启动苏州自主创新广场二期工程，大力发展科技服务业。推进知识产权示范城市和版权示范城市建设，发挥国家知识产权专利审查协作江苏中心的辐射带动作用，增强知识产权运用和保护能力。深化省科技金融结合试点工作，努力形成多元化、多层次、多渠道的科技投融资体系。做好质量强市工作，完善质量管理长效机制。统筹抓好人才队伍建设，坚持高端引领，突出产业导向，加大高层次、高技能、复合型人才培养和引进力度，实现创新人才培养引进同经济社会发展需求对接，创新项目同现实生产力对接，创新成果转化同产业对接。深入实施“姑苏人才”计划，办好国际精英创业周、苏州技能英才周、“赢在苏州”海外系列创业大赛等活动。改进人才管理，营造有利于人才辈出、人尽其才、才尽其用的政策环境。

　　2.坚持改革开放，增强持续发展动力。创新体制机制，破解发展难题，增创发展优势。加大行政体制改革力度。制定行政审批事项目录清单管理办法，做到目录之外无审批。推进行政审批服务中介机构改革，实现审批服务提速增效。深化商事登记制度改革，落实“先照后证”、注册资本认缴及企业年度报告公示等规定。以征信体系建设为基础和重点，加强事中事后监管，探索建立科学的抽查制度、责任追溯制度、经营异常名单和黑名单制度。实施预算管理制度改革，建立全面规范、公开透明的预算制度。继续推动政府部分职能向社会转移。加快事业单位分类改革，推进有条件的事业单位转为企业或社会组织；改革事业单位登记制度，取消事业单位年检。发展壮大市场主体。积极化解制约市场主体发展的各种束缚，使市场在资源配置中起决定性作用。以管资本为主加强国有资产监管，改革国有资本授权经营机制。扩大市属国资公司混合所有制改革试点范围，推动城建类国有企业以股权方式参与基础设施建设、生态环境建设、特许经营行业和古城保护等领域的投资。降低市场准入门槛，拓宽民间投资领域，促进民营经济健康发展。完成营业税改征增值税试点任务，进一步减轻纳税人负担。深化价格改革，引导产业结构调整和资源优化配置。支持金融改革创新。深入开展苏州工业园区外商投资企业外汇资本金管理方式改革，争取扩大试点范围，促进跨境投融资便利化。做大苏州工业园区跨境人民币创新业务，做好台资金融机构在昆山深化两岸产业合作试验区开展跨境人民币贷款试点工作。深化跨国公司总部外汇资金集中运营管理试点，拓宽企业参与面。鼓励民间资本发起设立民营银行、金融租赁公司等金融机构。确保苏州金融资产交易中心开业运营。深化开放型经济体制改革。主动对接中国（上海）自由贸易试验区，率先复制推广其成功经验。争创苏州工业园区国家开放创新综合改革试验区，积极开展综合保税区贸易多元化试点工作。加快建设昆山深化两岸产业合作试验区，加强两岸在人才、管理、金融、商贸、载体等方面的合作创新。推进外资项目快速审批试点、跨境电子商务服务试点、外贸综合服务体系建设等工作。争取获批开展国际贸易“单一窗口”试点，完善关检合作“一次申报、一次查验、一次放行”模式。支持张家港保税（港）区在汽车保税、汽车平行进口等方面探索实践。推动现有出口加工区转型为综合保税区，增强太仓港启运港退税功能。优化境外投资管理流程，实行以备案制为主的境外投资管理模式。拓展对外对内开放。突出国家级开发区的龙头带动作用，推动开发区发展由追求速度向追求质量转变，由政府主导向市场主导转变，由同质化竞争向差异化发展转变，由硬环境见长向软环境取胜转变。完善“苏州造”内外贸一体化网上销售平台，力争将常熟服装城、东方丝绸市场列入国家商务部内外贸结合商品市场试点。优化使用外资结构，着力引进高质量项目，服务业实际使用外资比重提高到38%，新兴产业和高技术项目实际使用外资比重提高到48%。坚持“引进来”、“走出去”并重，鼓励企业开展对外投资、境外并购以及建立境外营销网络、研发中心、服务外包接单服务中心，培育本土地标型跨国经营企业。积极投身“一带一路”和长江经济带建设，畅通“苏满欧”等贸易物流通道。规范外事管理，促进对外交流与合作。做好侨务、对台工作，服务经济社会发展。加强南北共建园区建设，继续抓好对口援建帮扶。

　　3.坚持统筹发展，提升城乡一体水平。以改革为动力、试点为抓手、政策为保障，在统筹城乡发展上取得更大进展。加快发展现代农业。按照产出高效、产品安全、资源节约、环境友好的要求，优化提升“四个百万亩”，着力构建现代农业体系。建设集中连片的高标准农田，新增现代农业园区面积6.67千公顷，新建和改建蔬菜基地267公顷。突出科技强农，提高农业物质装备和技术水平。发展农产品精深加工和现代营销，建设现代农产品物流园。保护农业种质资源，扩大本地特色优良品种种养规模。加强粮食生产基地建设，开展粮安工程国家级试点工作。鼓励发展合作社、家庭农场、龙头企业、专业大户等新型经营主体，建设新型职业农民和农民经纪人队伍，加快形成集约化、专业化、组织化、社会化的新型农业经营体系。强化农作物秸秆综合利用，实现秸秆资源化、商品化。深化城乡发展一体化综合改革。合理高效利用土地资源，以优化建设用地空间布局保障科学发展，以优化农业用地结构布局保护耕地资源，以优化镇村居住用地布局保障农民权益。全面开展农村土地承包经营权确权登记颁证工作。深化社区股份合作社股权固化改革，抓好“政经分离”改革试点。建设农村产权交易中心，推动农村产权流转交易公开、公正、规范运行。创新农村金融服务，发展农村普惠金融。实施富民强村工程。落实强农惠农富农政策，推动公共资源向农村倾斜，加快实现城乡基本公共服务均等化。开工新建100万平方米“一村二楼宇”物业载体，增加村级集体经济收入。落实集体经济薄弱村挂钩帮扶机制，提高村级稳定性收入。加强农村集体资产监督管理，保障农村合作经济组织规范健康发展。拓宽农民增收渠道，健全长效增收机制。实施农民安居工程，竣工交付安置房800万平方米。持续优化镇村面貌。完善镇村布局规划，编制实施重点村、特色村修建性详细规划，加强农村交通、电力、通讯、供水排水等基础设施建设。实施美丽城镇建设项目，提高城镇发展水平。深入推进“百村示范、千村整治”三年行动计划，新增美丽村庄示范点10个、三星级康居乡村100个，打造美丽村庄精品片区。积极开展被撤并老镇区的环境整治和长效管理。

　　4.坚持规划引领，优化城市功能品质。完善城市格局，增强承载能力，提升城市能级。高起点编制各类规划。推动新一轮苏州市城市总体规划和中心城区土地利用总体规划修编工作。编制中心城区控制性详细规划，制定城市综合交通、地下管线等专项规划。完善古城保护控制性详细规划、阊门历史文化街区保护规划、观前商圈规划，编制甪直、东山历史文化街区保护规划。推进“多规融合”，实现产业发展、城镇建设、土地利用、生态保护等规划有机衔接。高质量建设重大项目。继续建设东部综合商务城、西部生态科技城、南部太湖新城、北部高铁新城。积极配合沪通铁路苏州段建设。续建张家港疏港高速公路和常嘉高速公路昆山至吴江段，做好锡太高速公路苏州段前期准备工作。确保中环快速路及内环快速路延伸线建成通车，高标准改造城北路（原312国道城区段）。建设轨道交通2号线延伸线、3号线、4号线及支线和苏州高新区有轨电车2号线。完成苏南运河苏州段四级航道改三级航道整治工程，继续整治杨林塘、苏申内港线（吴淞江）航道。支持太仓港重点项目建设，做强做优现代港口物流。实施1000千伏特高压淮上线工程。开展电信网、广电网、互联网三网融合国家试点，落实“宽带中国”苏州实施方案，建设和完善4G 网络基础设施。新增人防设施95万平方米。高标准保护历史文化名城。古城是苏州历史文化的精华、不可再生的瑰宝，我们必须切实加强古城保护和有机更新，当好历史文化名城的守护者、建设者、传承人。加快构建分层次、分年代、分系列的保护体系，将物质和非物质文化遗存保护紧密结合起来，突出精细秀美特色，使苏州真正成为名副其实的历史文化保护示范区。以古城为核心，创新思路举措，实行整体保护、活态保护，精心实施平江历史文化片区成片保护利用、桃花坞历史文化片区保护整治、虎丘地区综合改造和古建老宅保护修缮等工程。促进古城、古镇、古村落保护与发展浑然一体，传承历史文脉，延续历史记忆，彰显江南水乡风貌，使这座被誉为“人间天堂”的名城生生不息、熠熠生辉。高效能加强城市管理。扎实推进“共建美丽新苏州、同享美好新生活”城市环境综合整治提升行动，集中整治一批长期影响城市形象的顽疾，落实长效管理措施，确保市容面貌得到新提升。继续改造市区人民路沿线街景容貌，抓好一批市政道路专项大修工程。推动“数字城管”信息共享，促进城市管理即时化、精细化、高效化。健全防灾减灾体系，提高防御应对自然灾害能力。完成全市地理国情普查工作。

　　5.坚持生态优先，营造良好人居环境。切实把生态文明建设放在更加突出的战略位置，始终不渝推动绿色发展、循环发展、低碳发展。加强生态建设。落实主体功能区制度和生态红线区域保护规划，坚守耕地保护、开发强度、生态保护“三条红线”。制定《苏州市生态补偿条例》实施细则。推进生态文明建设“十大工程”，深入开展“两河一江”、“两山一镇”环境综合整治，继续建设上方山石湖生态园和虎丘湿地公园。加快“绿色苏州”建设步伐，市区新增绿地430万平方米，抓好农村沿水、沿路、沿田、沿工业区绿化，优化绿化结构，提高绿化质量。重视森林抚育，强化森林防火基础设施建设。加强湿地保护，注重生态修复，创建湿地小区（公园）。狠抓环境治理。持之以恒实施大气污染防治和水环境治理。认真贯彻新《环境保护法》，落实源头保护、损害赔偿和责任追究制度，完善执法联动和长效监管机制，挂牌督办突出环境问题，严查重处违规排放、偷排偷放等行为。贯彻国务院制定的大气污染防治十条措施，突出抓好能源结构优化、产业结构调整、工业污染治理、绿色低碳交通、城市扬尘控制、应急预警提升六大工程，确保PM　浓度持续下降。推进太湖流域水环境综合治理、阳澄湖生态优化等工程，完成七浦塘综合整治主体工程，加强饮用水水源地保护。实施农村生活污水治理三年行动计划，完成1000个村庄生活污水治理工程。加强噪声污染防治以及固体废物、重金属、核与辐射环境监管，开展土壤污染调查与修复。完成七子山垃圾填埋场生态修复工程，健全建筑垃圾收集、运输、处理体系。落实病死畜禽无害化处理属地责任，建成各县级市、区无害化处理厂（场）。大力推进节能减排。严格执行污染物排放新标准，全面落实结构减排、工程减排、管理减排措施，开展火电、水泥、钢铁、平板玻璃等重点行业提标改造，狠抓工业废水、农业面源污染和机动车尾气排放治理，整治淘汰环保不达标企业。强化能源审计和节能精细化管理，推广应用节能新产品、新技术，实施节能环保产业化示范工程和LED照明示范工程。做好电力需求侧管理城市综合试点工作，开展企业节能低碳行动和“能效之星”创建活动。推进国家循环经济示范城市建设，争创国家级循环化改造示范试点园区。建设国家低碳试点城市，建立温室气体排放数据管理平台。积极开展排污权有偿使用和交易试点。

　　6.坚持惠民利民，增进人民群众福祉。按照习近平总书记提出的“努力让人民群众有更好的教育、更稳定的工作、更满意的收入、更可靠的社会保障、更高水平的医疗卫生服务、更舒适的居住条件、更优美的环境”的民生工作重点要求，优化民生保障体系，办好办实各项民生实事，让广大群众普遍得益受惠。促进就业创业。实行更加积极的就业政策，健全市场导向、城乡统筹的就业制度，全年新增就业15万人，苏州籍高校毕业生就业率保持在95%以上，确保“零就业”、“零转移”贫困家庭动态清零。降低创业门槛，支持自主创业。加快建设国家级创业孵化示范基地，扩大创业集聚效应。整合劳动力市场与人才市场，建立统一的人力资源市场，更好发挥中国苏州人力资源服务产业园“一园多区”的辐射带动作用。在充分就业、大众创业的基础上，不断提高城乡居民收入水平。完善社会保障、社会救助和养老服务。开展全民参保登记工作，注重统筹各类社会群体的保障待遇，争创社保更加公平可持续发展实验区。推进机关事业单位养老保险制度改革。鼓励符合条件的单位规范建立企业年金制度。采取更加有效的措施，以更大的力度帮扶困难群体，确保困难群体应保尽保、受助及时。完善社会医疗救助和大病保险制度，逐步扩大救助范围，提高救助水平。稳步提高城乡低保等救助标准，探索“救急难”工作由户籍人口向常住人口拓展。支持发展慈善事业和红十字事业，建立主动发现、快速响应、综合救助机制。对申请救助家庭的经济状况进行核对，确保社会救助对象认定准确公正。制定残疾人特殊困难救助办法，做好残疾人基本公共服务和残疾预防工作。稳步推进公办养老机构改革，引导和支持社会力量兴办养老机构，鼓励发展居家养老和社区养老服务，推动医疗卫生与养老服务融合发展。改善居民住行条件。完成省政府下达的年度保障性安居工程建设任务，推行住房保障货币化，提高租赁补贴标准，增加公租房供应量。继续推进梅巷片区、解放新村片区危旧房改造，加快实施城中村（无地队）整治。新增缴存住房公积金职工35万人，城镇职工住房公积金缴存覆盖率保持在85%以上。大力发展公共交通，优化线网结构，提高换乘效率；市区新购公交车550辆，其中新能源公交车250辆。改进城市交通管理，缓解道路拥堵现象。加强食品药品和市场物价监管。深入实施食品安全放心工程，开展餐饮环节食品安全专项整顿，扩大电子远程监管系统在学校食堂、集体用餐配送单位和大型餐饮单位的应用，完善肉菜流通追溯体系。严抓药品质量安全，强化对药品生产企业和高风险医疗器械的监管。加强对重要商品和服务价格的监测分析，及时防范和应对市场价格的异常波动。

　　7.坚持协调并进，推动社会和谐进步。加快发展社会事业，增强公共服务能力，维护社会公平正义。发展优质均衡适切的教育。优化教育设施布局，新建幼儿园16所、小学15所、初中10所。稳妥实施积分入学制度，保障流动就业人口随迁子女公平有序入学。完善中考制度，稳步实施招生考试评价制度改革，逐步形成体现素质教育要求、以学生发展为核心、具有苏州特色的教育综合评价体系。构建现代职业教育体系，深化校企合作、校产合作、校地合作。支持在苏高校加强内涵建设，促进名城名校融合发展。大力推动民办教育健康发展，不断满足多层次、多样化的教育需求。建设“学在苏州”市民终身学习云平台。促进文化繁荣。编制公共文化设施建设规划，做好国家公共文化服务标准化建设试点工作，打造城区“10分钟文化圈”、农村“十里文化圈”。建成市档案馆新馆、苏州现代传媒广场和妇女儿童活动中心迁建工程，建设苏州第二图书馆、苏州文博中心、苏州历史文化陈列馆、中国丝绸档案馆、苏州高新区文体中心、吴中区东吴文化中心。支持文艺创作，推动文艺创新，着力推出更多精品力作。加强古典园林保护和管理，完成可园修复一期工程。建设大运河世界文化遗产监测国家示范点，争取更多项目纳入国家“海上丝绸之路”和“江南水乡古镇”申遗方案。健全非物质文化遗产保护制度，公布首批市级生产性保护示范基地。推动健康事业发展。落实深化医药卫生体制改革省级试点任务，推进医疗、医保、医药联动，创新人事、薪酬等政策制度。完善医改财政补偿机制，公立医院全部实行药品零差率销售。探索实施执业医师在全市范围内集中注册、多点执业。建立基层首诊、分级诊疗、双向转诊的就医制度。启用苏州大学附属儿童医院园区总院，继续建设苏州大学附属第一医院主体迁建二期工程和市公共医疗中心、市第九人民医院、独墅湖医院、苏州科技城医院、姑苏医院。深入开展生育关怀主题活动，加强流动人口计划生育服务与管理。构建公共体育服务体系，续建苏州工业园区体育中心，改造运河体育主题公园，加快建设市民健身步道。办好第53届世界乒乓球锦标赛。创新和加强社会治理。践行社会主义核心价值观，弘扬张家港精神、昆山之路、园区经验“三大法宝”。深化文明城市长效建设，做好公共文明指数测评工作。开展民族团结进步创建活动，加强宗教事务管理。支持驻苏解放军和武警部队建设，做好国防动员和民兵预备役工作。深入开展拥军优属活动，争创全国双拥模范城“六连冠”。优化街道和社区管理体制，深化“政社互动”，健全以社区为基础、社会组织为载体、专业社会工作者为骨干的“三社联动”机制，促进多元善治。建成集社区管理服务、民意沟通、信息公开于一体的综合信息平台。实施流动人口积分管理制度。加强诚信社会建设，健全守信激励和失信惩戒机制，完善公共信用信息服务平台功能。依法规范信访秩序，推行信访网上受理，及时回应群众合理诉求，提高信访事项办理效能。构建立体化、现代化社会治安防控体系，严厉打击暴力恐怖、涉黑犯罪、邪教和黄赌毒等违法犯罪行为，高度警惕和严密防范敌对势力的分裂渗透颠覆活动，有效管控化解影响社会稳定的问题。做好社区矫正和刑释人员安置帮教工作，加强特殊人群管理服务。完善人民调解、行政调解、司法调解联动工作体系，积极化解社会矛盾。狠抓安全生产工作。认真贯彻新《安全生产法》，牢固树立安全发展理念，始终把人民群众生命财产安全放在首位，不断强化“红线”意识和“底线”思维，督促企业严格落实安全生产主体责任。严格执行“党政同责、一岗双责、齐抓共管”的规定，坚持“管行业必须管安全、管业务必须管安全、管生产经营必须管安全”和“分级负责、属地为主”的原则，不折不扣落实属地监管、综合监管、直接监管的各项责任，切实防范重特大事故。健全隐患排查治理长效机制，深化“打非治违”专项行动，坚决堵塞漏洞、排除隐患。抓好监管队伍建设，夯实监管基层基础。加快企业安全生产标准化建设，优化重点企业、高危企业信息化监管系统，提高安全生产监管智能化水平。完善应急预案，落实物资装备，加强应急联动，提高快速处置能力。

　　8.坚持依法行政，推进法治政府建设。紧紧围绕使法治成为苏州核心竞争力的重要标志和内容这一要求，加快建立权责统一、权威高效的依法行政体制，建设职能科学、权责法定、执法严明、公开公正、廉洁高效、守法诚信的法治政府。依法履行政府职能。严格按照法定权限和法定程序履行责任、行使权力，切实做到法定职责必须为、法无授权不可为。修订苏州市法治政府建设指标体系，制定法治政府建设规划（2016～2020）。认真执行重大行政决策程序规定，严格落实公众参与、专家论证、风险评估、合法性审查、集体讨论决定等法定程序，确保决策制度科学、程序正当、过程公开、责任明确。强化决策执行的过程监督和反馈纠偏，建立重大决策终身责任追究制度及责任倒查机制。完善政府规章、规范性文件制定程序，拓宽公众参与政府立法渠道。推动政府工作部门和镇政府、街道办事处建立法律顾问制度。提高行政执法水平。落实执法责任，规范执法行为，做到严格规范公正文明执法。深化行政执法体制改革，减少市、县级市（区）两级政府执法队伍种类，推行跨部门综合执法，开展镇级综合执法试点。推动县级市、区城市管理领域相对集中行政处罚权工作向建制镇延伸。加大关系群众切身利益的重点领域执法力度，确保有法必依、执法必严、违法必究。认真落实行政执法调查取证、全过程记录等规定，严格执行重大执法决定法制审核制度，推行行政执法公示。细化、量化行政裁量标准，规范裁量范围、种类和幅度。加强权力监督制约。做好行政应诉工作，推行行政机关负责人出庭应诉。推进行政复议规范化建设，开展行政复议委员会试点和相对集中行政复议权试点。健全行政权力公开透明运行机制，坚决查处行政不作为、乱作为行为，切实纠正损害群众利益的不正之风。深入开展执法监察、廉政监察、效能监察，加大行政问责力度。创新审计方式，提高审计效率，加强对重大决策部署落实情况的跟踪审计，推进公共资金、国有资产、国有资源和领导干部经济责任履行情况的审计监督全覆盖。坚持以公开为常态、不公开为例外原则，全面深化政务公开，重点做好财政预决算、公共资源配置、重大建设项目批准和实施、社会公益事业建设等领域的信息公开工作。自觉接受人民代表大会及其常委会的法律监督、工作监督，接受人民政协的民主监督，接受司法机关的监督，接受新闻舆论和社会公众的监督，认真实施人大议案，办理代表建议和政协提案。推动法治社会建设。总结发掘苏州文化精髓，做到弘扬优秀传统文化与推进法治苏州建设相互融合。健全普法宣传教育机制，实行“谁执法、谁普法”的普法责任制，提高普法实效，争创全国“六五”普法先进市。深入开展政府学法活动，增强运用法治思维和法治方式深化改革、推动发展、化解矛盾、维护稳定的能力。创新法治惠民举措，推进县级市（区）、镇（街道）公共法律服务中心和村（社区）便民法律服务站建设，构建覆盖城乡的公共法律服务体系。完善法律援助制度，扩大援助范围。推动法律服务队伍建设，促进律师、公证等法律服务业发展。持续开展法治城市、法治县（市、区）和基层民主法治村（社区）创建活动，深化基层组织和部门、行业依法治理，支持各类社会主体自我约束、自我管理。强化法律在维护群众权益、化解社会矛盾中的权威地位，引导和支持群众理性表达诉求、依法维护权益。

　　9.坚持从严治政，建设人民满意政府。深入学习贯彻习近平总书记系列重要讲话精神，着力在武装头脑、坚定理想信念上下功夫，在把握要旨、融会贯通上下功夫，在指导实践、推动工作上下功夫，始终在思想上、政治上、行动上与以习近平同志为总书记的党中央保持高度一致。落实新形势下坚持从严治党的要求，严格执行党建工作责任制，切实做到党建工作和中心工作一起谋划、一起部署、一起考核，坚决防止“一手硬、一手软”，全面提升政府系统党建水平。深入推进作风建设。巩固拓展党的群众路线教育实践活动成果，按照中央巡视组反馈意见，对照群众意见建议，以“钉钉子”精神抓好整改落实，确保事事有着落、项项见成效。以锲而不舍、驰而不息的决心，踏石留印、抓铁有痕的劲头，持之以恒反“四风”、改作风，实现作风建设的常态化、长效化。坚决执行改进工作作风、密切联系群众的各项规定，认真落实中央和省委、市委部署开展的专项治理任务，确保不留死角、不留盲区、不留空白。加强政风行风建设，切实解决群众反映强烈的突出问题，真正做到勇于担当、敢于负责、善抓落实。扎实开展领导干部下基层“三访三促”活动，更好服务挂钩企业、重点项目和帮扶对象，积极回应群众诉求，密切同群众的血肉联系。完善网上办事大厅和实体大厅“线上线下、虚实一体”的政务服务平台，探索推行政务服务中心与便民服务中心、公共资源交易中心一体化运行管理模式。厉行勤俭节约。严格遵守《党政机关厉行节约反对浪费条例》，狠刹奢靡之风，严禁浪费行为。规范和改进公务接待、商务接待工作，改革公务用车制度，加强因公出国（境）管理，继续压缩“三公”经费支出。大力压减会议，精简文件简报资料，严控创建达标检查评比和节庆论坛等活动，禁止使用财政性资金举办年会、经营性文艺演出。加大反腐倡廉力度。严格落实党风廉政建设责任制，健全政府系统惩治和预防腐败体系，坚持有腐必反、有贪必肃、违规必纠，始终保持惩治腐败的高压态势。对违反纪律的行为，发现一起，查处一起，绝不姑息、绝不手软。

　　编制“十三五”规划是今年的一项重要工作。我们要以习近平总书记对江苏发展提出的最新定位、最新要求和“两个百年”的奋斗目标为行动指南，认真总结苏州发展的经验和特征，以全球视野、战略眼光，把准重大目标、重大课题、重大布局，凝聚各方智慧，科学谋划未来，精心编制适应时代要求、符合苏州实际、反映人民意愿的高质量规划。

　　各位代表，今年改革发展任务艰巨而繁重，做好各项工作需要倍加努力、久久为功。让我们紧密团结在以习近平同志为总书记的党中央周围，在中共苏州市委的坚强领导下，齐心协力、真抓实干，迎难而上、开拓进取，在“迈上新台阶、建设新江苏”的进程中走在最前列，努力谱写好中国梦的苏州篇章！
